# Interaction of N_2_, O_2_ and H_2_ Molecules with Superalkalis

**DOI:** 10.1002/open.202300253

**Published:** 2024-01-09

**Authors:** Harshita Srivastava, Ambrish Kumar Srivastava, Neeraj Misra

**Affiliations:** ^1^ Department of Physics Deen Dayal Upadhyaya Gorakhpur University 273009 Gorakhpur Uttar Pradesh India; ^2^ Department of Physics University of Lucknow 226007 Lucknow Uttar Pradesh India

**Keywords:** Superalkalis, *ab initio* calculation, reduction, adsorption, diatomic molecules

## Abstract

Superalkalis (SAs) are exotic clusters having lower ionization energy than alkali atoms, which makes them strong reducing agents. In the quest for the reduction of diatomic molecules (X_2_) such as N_2_, O_2_, and H_2_ using Møller‐Plesset perturbation theory (MP2), we have studied their interaction with typical superalkalis such as FLi_2_, OLi_3_, and NLi_4_ and calculated various parameters of the resulting SA−X_2_ complexes. We noticed that the SA−O_2_ complex and its isomers possess strong ionic interaction, which leads to the reduction of O_2_ to O_2_^−^ anion. On the contrary, there are both ionic and covalent interactions in SA−N_2_ complexes such that the lowest energy isomers are covalently bonded with no charge transfer from SA. Further, the interaction between SA and H_2_ leads to weakly bound complexes, which results in the adsorption of H_2_ molecules. The nature of interaction is found to be closely related to the electron affinity of diatomic molecules. These findings might be useful in the study of the activation, reduction, and adsorption of small molecules, which can be further explored for their possible applications.

## Graphical abstract

N_2_ (blue), O_2_ (red) and H_2_ (grey) interact differently with superalkali clusters. Their interaction varies from strong covalent to weak van der Waals interaction leading to the possibility of reduction or adsorption of these small molecules.

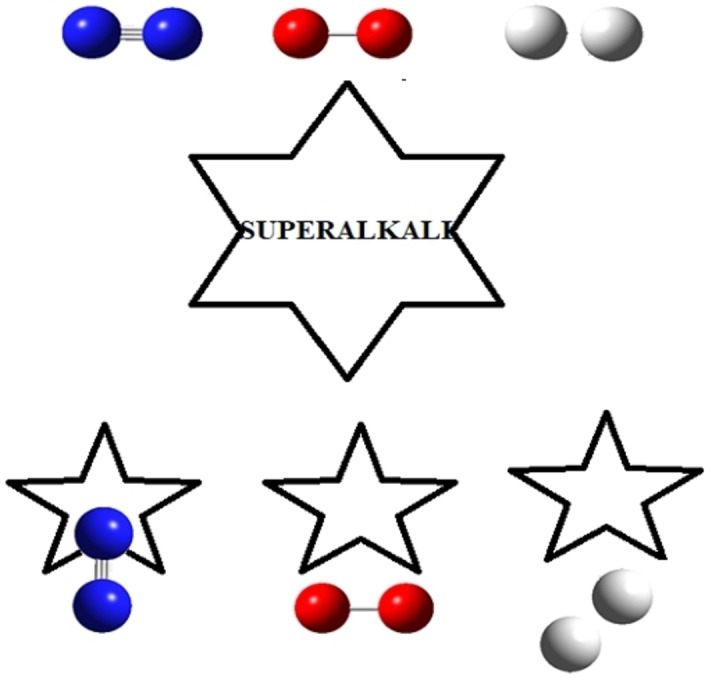

## Introduction

Over the last four decades, superalkalis have become one of the most attractive topics of research. These exotic clusters are characterized by lower ionization energies (IEs) than those of alkali‐metal atoms (5.39–3.89 eV).^[1]^ Superalkalis are hyperalkalized species of the ML_*k*+*n*_ type (where L is an alkali metal atom, *k* is the maximal formal valence of the central atom M, and *n*≥1).[^2^, ^3^, ^4^, ^5^, ^6^, ^7^] Li_3_O, which was experimentally identified by Wu et al.^[8]^ and subsequently revealed to be highly thermodynamically stable towards dissociation or loss of an electron by Schleyer et al.,^[9]^ is a typical example of this sort of superalkali cluster. Since then, several other superalkalis have been explored in their various forms.[^10^, ^11^, ^12^, ^13^, ^14^, ^15^, ^16^, ^17^, ^18^] The aromatic superalkalis have also been reported recently.[^19^, ^20^, ^21^] The application of superalkalis includes the design of nonlinear optical (NLO) by the doping of superalkalis.^[22]^ Recently, Al_12_N_12_ nanocages doped with superalkalis (Li_2_F and Li_3_F) have been developed and theoretically explored to clarify the relationships between geometric, electronic, and nonlinear optical features.^[23]^ Another group of researchers explored an improved NLO material by doping superalkalis into adamantanes.^[24]^ The low IE of superalkalis can be exploited to reduce and activate several stable molecules. Reportedly, the superalkali species are employed to activate small molecules such as carbon monoxide,^[25]^ dinitrogen (N_2_),^[26]^ NO_x_,^[27]^ etc. More importantly, it can be used for the reduction of the CO_2_ molecule which possesses no positive electron affinity.[^28^, ^29^, ^30^, ^31^]

Nitrogen (N_2_) is earth's most prevalent gas molecule whose inertness makes it impossible to employ directly for metabolism.^[32]^ Its high ionization energy (15 eV) and its inability to attach an extra electron provide formidable obstacles to its oxidation and reduction, respectively.^[33]^ To find more productive and effective strategies to activate N_2_, several studies have been conducted.[^32^, ^33^, ^34^, ^35^, ^36^, ^37^] It was reported that dinitrogen (N_2_) can also be activated by a binuclear superalkali Li_3_F_2_.^[26]^ Likewise, there are sizable kinetic hurdles related to the activation of oxygen (O_2_), even if its reduction is exergonic.^[37]^ As a result, catalysts that may mediate the physiological reduction of O_2_ are necessary to promote its reactivity. Nowadays, a large percentage of oxygen reduction catalysts utilized in the industry makes use of precious metals like platinum and palladium.[^39^, ^40^, ^41^] These metals are not suitable for long‐term industrial applications due to their limited availability. Most interesting is the case of hydrogen (H_2_). It is the most intriguing form of energy for the future. Nevertheless, a safe and effective storage method must be developed before hydrogen can be employed on a big scale, particularly for on‐board vehicle applications.[^42^, ^43^, ^44^] Materials‐based storage, which can be separated into adsorbent and chemical storage, is a better strategy in terms of safety, clean energy, and hydrogen storage capacity.[^45^, ^46^] Both theoretical and experimental studies have shown that coating nanomaterials with metals, especially alkali metals (AMs) can significantly boost hydrogen absorption.[^47^, ^48^, ^49^, ^50^] It has been reported recently that Ni@Al_12_P_12_, Ni@B_12_P_12_, and Co@B_12_P_12_ systems have good stability, and are good candidates as excellent catalysts for hydrogen evolution reaction (HER).^[51]^ It was found that among first‐row transition metals (Fe−Zn) decorated magnesium oxide nanocages (TM@Mg_12_O_12_), Fe@Mg_12_O_12_ possesses a very low activation barrier, and thus catalyzes the HER reaction.^[52]^

In this study, we have studied the interaction of diatomic molecules such as N_2_, O_2_, and H_2_ with superalkali species, FLi_2_, OLi_3_, and NLi_4_ using Møller‐Plesset perturbation theory (MP2). This study is expected to provide some new insights into the activation, reduction, and capture of small molecules by exotic clusters.

## Results and Discussion

Before we discuss the interaction of these molecules with superalkalis, we first take isolated diatomic molecules. We have also collected the calculated and experimental bond lengths as reported in the literature[^57^, ^58^, ^59^] in Table 1. One can see that our method is capable of reproducing bond length within the limit of 0.03 Å. The electron affinities of the N_2_, O_2,_ and H_2_ are either very low or negative suggesting their inability to attach an electron. We have compared their electron affinities with corresponding experimental values.[^60^, ^61^, ^62^] One can also notice that the extra electron leads to an increase in the bond lengths of N_2_ by 0.03 Å, O_2_ by 0.03 Å, and H_2_ is increased by 0.02 Å.

**Table 1:** Bond length and electron affinity of N_2_, O_2,_ and H_2_. Experimental values of bond length are taken from (Ref. [57–59]) and electron affinity from (Ref. [60–62]).

X_2_	Bond length (in Å)		Electron affinity (in eV)	
	Calculated	Experimental	Calculated	Experimental
N_2_	1.12	1.09	−2.29	−1.6
O_2_	1.24	1.21	1.47	1.07±0.007
H_2_	0.76	0.74	−1.88	−2.0

### Interaction of N_2_ with superalkalis

**Figure 1:**
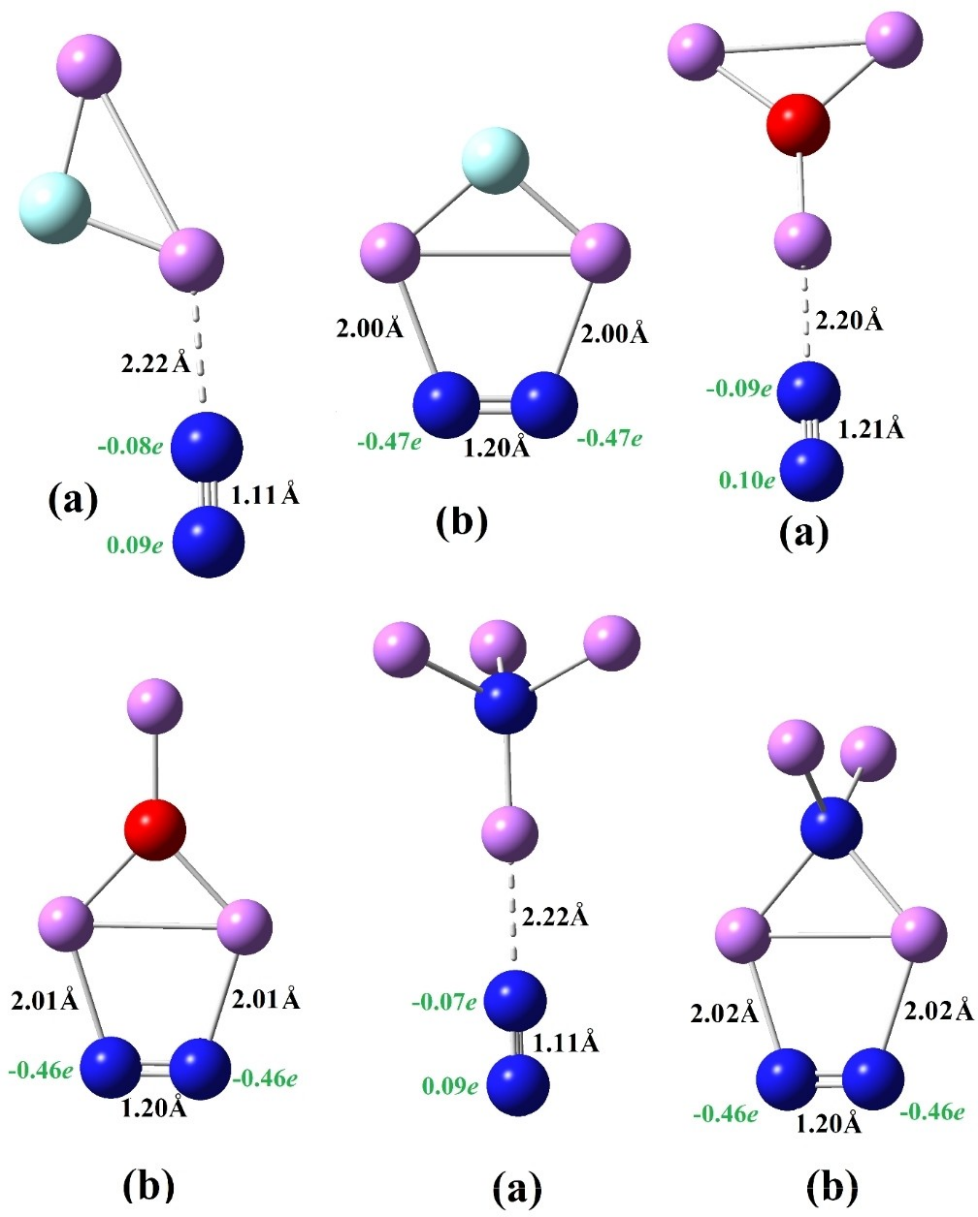
Equilibrium structures of SA−N_2_ (FLi_2_, OLi_3_ and NLi_4_) and their isomers.

We first discuss the interaction of SA with N_2_, which forms SA−N_2_ complexes for SA=FLi_2_, OLi_3,_ and NLi_4_ as shown in Figure 1, and corresponding parameters are listed in Table 2. We obtained two geometries for the FLi_2_−N_2_ complex in which (a) is lower in energy than (b) by 0.03 eV such that the N_2_ moiety interacts with one Li atom and the two Li atoms of FLi_2_ in (a) and (b), respectively. There are two isomers of the OLi_3_−N_2_ complex also shown in Figure 1. In the OLi_3_−N_2_ (a) isomer, one N of the N_2_ moiety interacts with the single Li atom of the OLi_3_ superalkali, similar to FLi_2_−N_2_ (a). The second isomer OLi_3_−N_2_ (b) is 0.22 eV higher in energy where both atoms of N_2_ interact with the two Li atoms of OLi_3_ similar to FLi_2_−N_2_ (b). Similarly, there are two isomers of the NLi_4_−N_2_ complex like FLi_2_−N_2_ by 0.16 eV apart. In all these SA−N_2_ complexes, the most stable isomer corresponds to the structure in which the interaction takes place between a single Li atom of OLi_3_ and an N atom of N_2_ molecule.

**Table 2:** Relative energy (Δ*E*_r_) bond length, binding energy (Δ*E_b_*), ionic dissociation energy(Δ*E_d_*), charge transferred to X_1_ and X_2_ (where X=N, O and H) and the net charge (*Q*) on N_2_, O_2_ and H_2_ molecules by using MP2/6‐311++G(3df,p) scheme.

System	Conformers	Δ*E_r_* (eV)	Bond Length X−X (Å)	Δ*E_b_* (eV)	Δ*E_d_* (eV)	$Q_{X_{1}}\;$ (*e*)	$Q_{X_{2}}$ (*e*)	*Q* (*e*)
FLi_2_−N_2_	(a)	0	1.11	0.19	2.48	−0.08	0.09	0.01
	(b)	0.03	1.19	0.16	2.45	−0.47	−0.47	−0.94
OLi_3_−N_2_	(a)	0	1.11	0.24	2.53	−0.09	0.10	0.01
	(b)	0.22	1.19	0.03	2.31	−0.46	−0.46	−0.92
NLi_4_−N_2_	(a)	0	1.11	0.19	2.48	−0.07	0.09	0.02
	(b)	0.16	1.20	0.03	2.25	−0.46	−0.46	−0.92
FLi_2_−O_2_	(a)	0	1.35	4.37	2.89	−0.47	−0.47	−0.94
	(b)	0.55	1.30	3.81	2.33	−0.82	−0.14	−0.96
	(c)	0.73	1.35	3.63	2.15	−0.47	−0.47	−0.94
OLi_3_−O_2_	(a)	0	1.34	4.21	2.73	−0.47	−0.47	−0.94
	(b)	0.55	1.31	3.65	2.30	−0.78	−0.17	−0.95
NLi_4_−O_2_	(a)	0	1.35	3.91	2.45	−0.45	−0.45	−0.90
	(b)	0.31	1.30	3.61	2.20	−0.76	−0.18	−0.94
FLi_2_−H_2_	(a)	0	0.74	0.07	1.96	0.02	−0.01	0.01
	(b)	0.04	0.74	0.03	1.91	0.02	−0.03	−0.01
OLi_3_−H_2_	–	–	0.74	0.10	1.98	0.03	0.03	0.06
NLi_4_−H_2_	–	–	0.74	0.05	1.94	0.04	0.01	0.05

Table 2 shows the calculated binding energies (Δ*E_b_*) for each complex along with their isomers. The calculated binding energy for FLi_2_−N_2_ (a), OLi_3_−N_2_ (a) and NLi_4_−N_2_ (a) is 0.16, 0.24, and 0.19 eV, respectively. These values indicate that the resultant complexes SA−N_2_ are strongly bound and highly stable. We have also calculated the ionic dissociation energy (Δ*E_d_*) of the SA−N_2_ complexes by using equation 2 and listed in Table 2. The dissociation energies of the most stable isomers FLi_2_−N_2_ (a), OLi_3_−N_2_ (a), and NLi_4_−N_2_ (a) are 2.48, 2.53, and 2.48 eV, respectively. The Δ*E_d_* values of all these complexes and their isomers follow the same trend as those of their Δ*E_b_* values.

In order to understand the interaction, i. e., the nature of bonding in the SA−N_2_ complex, we have also listed NPA charges on each N atom and whole N_2_ moiety in Table 2. It is found that the bonding between FLi_2_ and N_2_ is covalent in FLi_2_−N_2_ (a) whereas it is ionic in FLi_2_−N_2_ (b). On the contrary, the bond between OLi_3_ and N_2_ covalent in the (a) but ionic in OLi_3_−N_2_ (b). Similarly, the NLi_4_−N_2_ (a) is covalently bonded whereas in NLi_4_−N_2_ (b), the interaction is ionic. Therefore, the lowest energy structures of all SA−N_2_ complexes are stabilized by covalent interactions. The calculated bond length of the N_2_ moiety in all these lowest energy isomers is 1.11 Å, which is almost equal to that of the N_2_ molecule (see Table 1). On the contrary, the bond length of N_2_ moiety in higher energy isomers is increased to 1.20 Å due to electrostatic repulsion, leading to the reduction of N_2_ molecule. This is inconsistent with the dramatic elongation of the N−N bond reported on the activation of N_2_ through (Li_3_F_2_)_*n*_N_2_ (*n*=1–6).^[23]^

### Interaction of O_2_ with superalkalis

**Figure 2:**
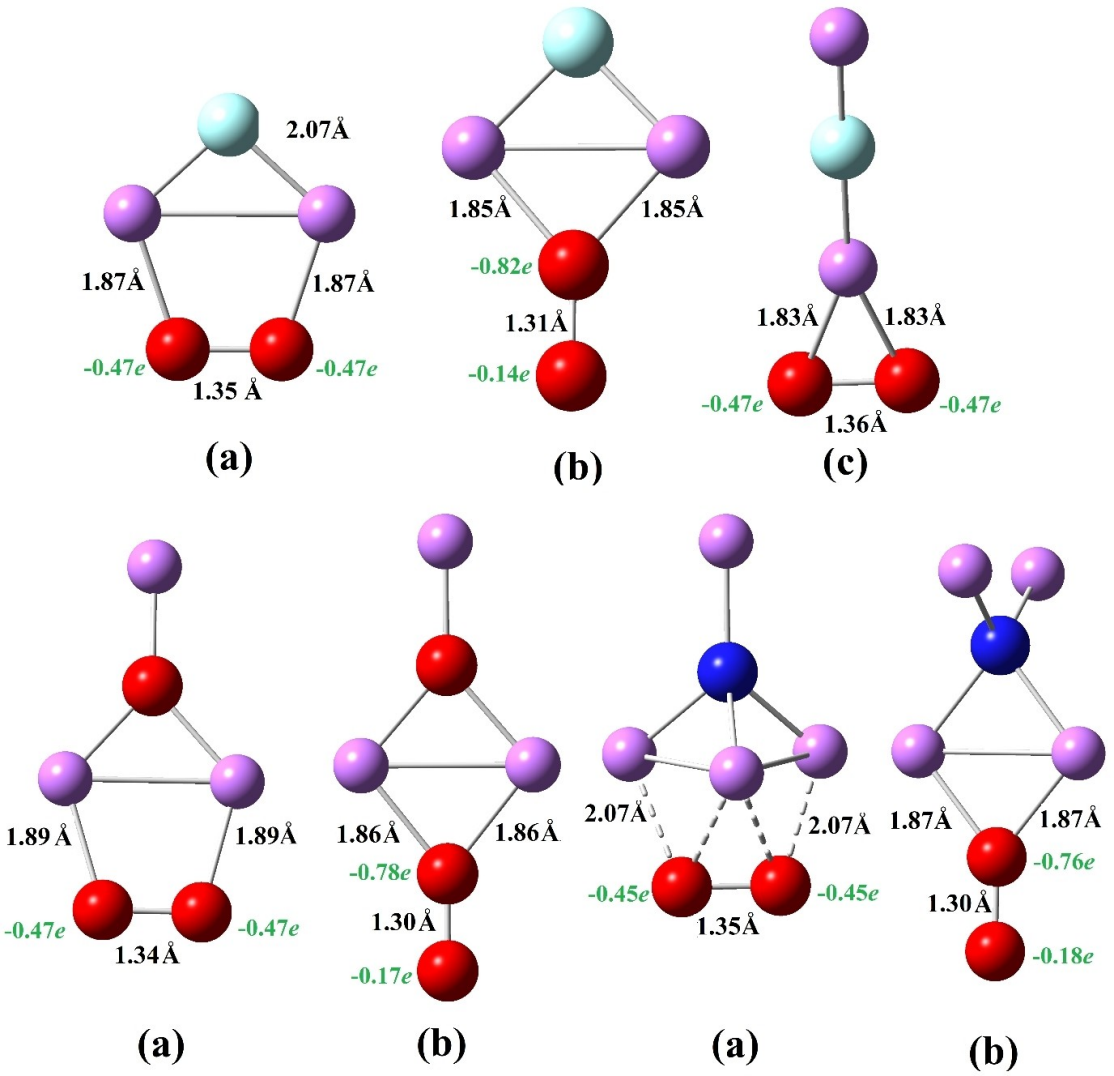
Equilibrium structures of SA−O_2_ (FLi_2_, OLi_3_ and NLi_4_) and their isomers.

The interaction of SA with the O_2_ molecule leads to SA−O_2_ complexes for SA= FLi_2_, OLi_3_, and NLi_4_ whose optimized structures are shown in Figure 2. We have obtained three isomers (a), (b) and (c) for FLi_2_−O_2_. In the FLi_2_−O_2_ (a) configuration, both atoms of the dioxygen (O_2_) interact through the two Li‐atoms of the FLi_2_ superalkali cluster whereas in FLi_2_−O_2_ (b), which is 0.55 eV higher in energy, only one O atom interacts with the two Li‐atoms. In the third isomer FLi_2_−O_2_ (c) having 0.73 eV higher energy, the interaction takes place between a single O atom of the O_2_ moiety and the Li atom of FLi_2_. Similarly, the lowest energy of OLi_3_−O_2_ and NLi_4_−O_2_ complexes corresponds to the structures (a) in which both O atoms of O_2_ moiety interact with OLi_3_ or NLi_4_ superalkali and (b) isomers are 0.55 and 0.31 eV higher in energy for OLi_3_ and NLi_4_, respectively.

The binding energies (Δ*E_b_*) of SA−O_2_ complexes are also calculated as listed in Table 2. The binding energy of the lowest energy structures, FLi_2_−O_2_ (a), OLi_3_−O_2_ (a), and NLi_4_−O_2_ (a) are 4.37, 4.21, and 3.91 eV, respectively. The order of binding energies of SA−O_2_ complexes becomes FLi_2_−O_2_ (a) > OLi_3_−O_2_ (a) > NLi_4_−O_2_ (a), which is similar to SA‐SO_2_ complexes.^[63]^ The ionic dissociation energy (Δ*E_d_*) of these complexes is also listed in Table 2. One can notice that the Δ*E_d_* of the most stable isomers are 2.90 eV for FLi_2_−O_2_, 2.73 eV for OLi_3_−O_2,_ and 2.45 eV for NLi_4_−O_2_, which is in accordance with the order of Δ*E_b_* values of these complexes.

The net charge (*Q*) on dioxygen for each of the SA−O_2_ complexes is calculated to better understand the interaction of O_2_ and superalkalis. The most stable structures, namely, FLi_2_−O_2_ (a), OLi_3_−O_2_ (a), and NLi_4_−O_2_ (a) acquired the *Q* as 0.95*e*, 0.94*e*, and 0.90*e*, respectively. Therefore, the bond between SA and O_2_ is strangely different from the bond nature of SA−N_2_, discussed in the previous section. Based on the charge transfer, the bond between SA and O_2_ is ionic for all SA−O_2_ isomers, however, the charge distribution on O atoms of the O_2_ moiety is unequal in (b) isomers (see Table 2). A slight increase in the bond distance of O−O of the O_2_ moiety is also observed. For instance, the calculated O−O bond distance acquired the approximate value of 1.35 Å in each most stable SA−O_2_ complex, which is equivalent to that of O_2_ anion.^[58]^ Thus, the charge transfer and geometry of SA and O_2_ complexes may suggest that superalkalis clusters can be found to be useful in the reduction of O_2_.

### Interaction of H_2_ with superalkalis

**Figure 3:**
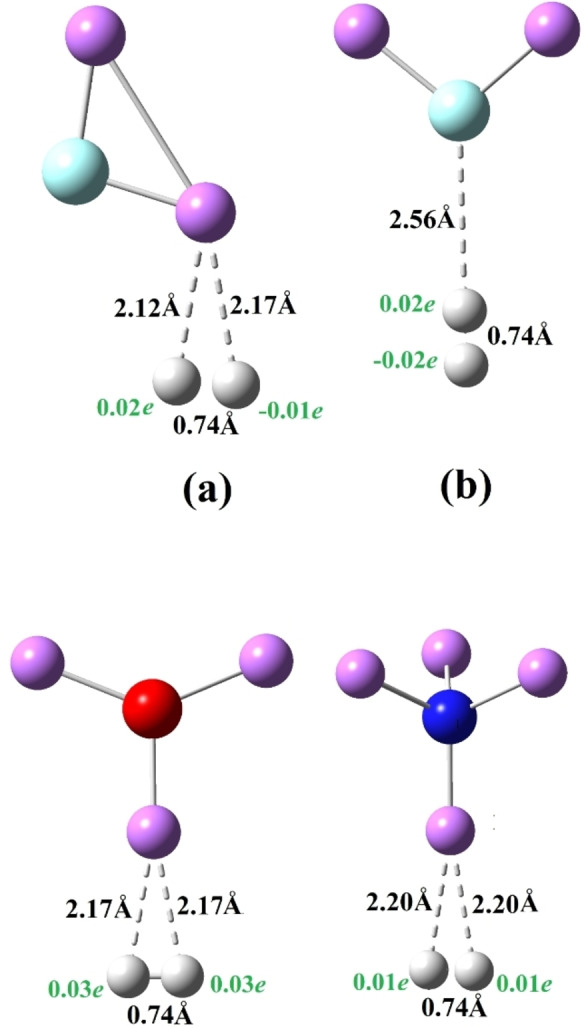
Equilibrium structures of SA−H_2_ (FLi_2_, OLi_3_ and NLi_4_) and their isomers.

Finally, we studied the interaction of dihydrogen (H_2_) with SA leading to SA−H_2_ complexes as shown in Figure 3 and the corresponding parameters are listed in Table 2. In the lowest energy isomer, both H‐atoms of H_2_ interact with the single Li‐atom of the superalkali (SA) clusters. The binding energy (Δ*E_b_*) values of these SA−H_2_ complexes are listed in Table 2. One can see that these complexes are weakly bound such that the Δ*E_b_* of the lowest energy isomer of the FLi_2_−H_2_ (a) complex is calculated to be 0.07 eV. However, the Δ*E_b_* of the OLi_3_−H_2_ and NLi_4_−H_2_ complex are 0.10 and 0.05 eV respectively indicating weak interaction between SA and H_2_ at all (see Figure 3). The ionic dissociation energy (Δ*E_d_*), listed in Table 2, shows that there is an energy barrier of 1.96±0.02 V, which makes it stable against dissociation to ionic fragments.

The charge on the H_2_ moiety in the most stable isomers, is very close to zero, limited to 0.06*e*. Therefore, the interaction between SA and H_2_ is entirely different. This interaction tends to polarize the H_2_ molecule irrespective of the nature of SA, which eventually leads to weakly bound complexes. The H−H bond length of 0.74 Å in SA−H_2_ complexes also indicates that the interaction does not affect the H_2_ molecule. This may suggest the adsorption of H_2_ molecule by superalkalis.

## Conclusions

We have investigated the structures of the SA−X_2_ complexes formed by the interaction of superalkalis (SAs) such as FLi_2_, OLi_3,_ and NLi_4_ with diatomic molecules (X_2_; X=N, O, and H) and their possible isomers. We have analyzed various parameters like relative energy, binding energy, ionic dissociation energy, bond length, and charge transfer between SA and X_2_. All these complexes are found to be stable due to positive binding energy and dissociation energy values. The binding energies of SA−O_2_ are found to be much higher as compared to SA−N_2_ and SA−H_2_. This can be expected due to charge transfer from SA to the O_2_ molecule, leading to the ionic SA−O_2_ complexes. In the case of SA−N_2_, the ionic complexes are higher in energy and there is no appreciable charge transfer from SA to N_2_ molecule in the lowest energy structures. Thus, SA−N_2_ complexes are more likely to be covalently bonded. Even more interesting is the case of SA−H_2_ in which the interaction between SA and H_2_ is very weak such that the H_2_ molecule maintains its identity. Therefore, the SA tends to reduce the O_2_ molecule but not H_2_. This can be expected due to no positive electron affinities of N_2_ and H_2_, in contrast to O_2_. Thus, the findings of the study suggest the interaction of small molecules with superalkalis may lead to reduction as in the case of O_2,_ and adsorption as in the case of H_2,_ or both as in the case of N_2_. The study may also shed some light on the possible application of superalkalis for hydrogen storage.

## Computational Section

The equilibrium structure of FLi_2_−X_2_, OLi_3_−X_2_, and NLi_4_−X_2_ (X= N, O, and H) and their isomers was obtained through geometry optimization at MP2 method^[53]^ using 6‐311++G (3df,p) basis set in Gaussian 16 program.^[54]^ The visualization of molecular structure trajectories was done by the GaussView program.^[55]^ These optimized structures correspond to at least some local minima because the vibrational harmonic frequencies were found to be positive. We have calculated the binding energy (BE) of these structures by using equation 1 which is given as:
(1)

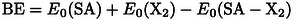




To further analyze the stability, dissociation energy (Δ*E*_d_) against ionic fragments is obtained by using equation 2:
(2)

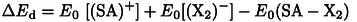




where *E*_0_(..) represents the total electronic energy of respective species and ZPVE. We have calculated the net charge (*Q*) on X_2_ and charges on individual X atoms with the help of the natural bond orbital (NBO) method^[56]^ as available in the Gaussian 16 program.

## Author Contributions

H. S.: literature survey, calculations, data collection, and writing draft. A. K. S.: conceptualization, supervision, editing, and finalizing the draft.

## Conflict of interests

The authors declare no conflict of interest.

1

## Data Availability

Data associated with this article will be available upon reasonable request.

## References

[1] S. G. Lias , J. E. Bartmess , J. F. Liebman , J. L. Homes , R. D. Levin , W. G. J. Mallard , Phys. Chem. Ref. Data 1988, 1, 17.

[2] G. L. Gutsev , A. I. Boldyrev , Chem. Phys. Lett. 1982, 92, 262.

[3] G. L. Gutsev , A. I. Boldyrev , Adv. Chem. Phys. 1985, 61, 169.

[4] V. G. Zakrzewski , W. v. Niessen , A. I. Boldyrev , P. v. R. Schleyer , Chem. Phys. 1993 174, 167.

[5] A. N. Alexandrova , A. I. Boldyrev , J. Phys. Chem. A 2003, 107, 554.

[6] S. R. Velickovic , V. J. Koteski , J. N. B. Cavor , V. R. Djordjevic , J. M. Cveticanin , J. B. Djustebek , M. V. Veljkovic , O. M. Neskovic , Chem. Phys. Lett. 2007, 448, 151.

[7] E. Rehm , A. I. Boldyrev , P. v. R. Schleyer , Inorg. Chem. 1992, 31, 4834.

[8] C. H. Wu , H. Kudo , H. R. Ihle , J. Chem. Phys. 1979, 70, 1815.

[9] P. v. R. Schleyer , E. U. Wuerthwein , J. A. Pople , J. Am. Chem. Soc. 1982, 104, 5839.

[10] J. Tong , Y. Li , D. Wu , Z. R. Li , X. R. Huang , J. Phys. Chem. A 2011, 115, 2041. 21332234 10.1021/jp110417z

[11] N. Hou , Y. Li , D. Wu , Z.-R. Li , Chem. Phys. Lett. 2013, 575, 32.

[12] T. Zhao , Q. Wang , P. Jena , Nanoscale 2017, 9, 4891. 28247884 10.1039/c7nr00227k

[13] A. K. Srivastava , Mol. Phys. 2018, 116, 1642.

[14] W.-M. Sun , Y. Li , D. Wu , Z.-R. Li , J. Phys. Chem. C 2013, 117, 24618.

[15] S. Giri , G. N. Reddy , P. Jena , J. Phys. Chem. Lett. 2016, 7, 800. 26882875 10.1021/acs.jpclett.5b02892

[16] X. Liu , Y. Song , W. Geng , H. Li , L. Xiao , W. Wu , Catalysts. 2016, 6, 75.

[17] A. K. Srivastava , N. Misra , Chem. Phys. Lett. 2015, 639, 307.

[18] S. Giri , S. Behera , P. Jena , J. Phys. Chem. A 2014, 118, 638. 24383446 10.1021/jp4115095

[19] A. K. Srivastava , Mol. Phys. 2020, 118, e1730991.

[20] A. K. Srivastava , Mol. Phys. 2018, 116, 1642.

[21] A. K. Srivastava , N. Misra , Mol. Phys. 2014, 112, 2621.

[22] R. Bano , M. Asghar , K. Ayub , T. Mahmood , J. Iqbal , S. Tabassum , R. Zakaria , M. A. Gilani , Front. Mater. 2021, 8, 783239.

[23] R. Bano , M. Arshad , T. Mahmood , K. Ayub , A. Sharif , S. Tabassum , M. A. Gilani , Mater. Sci. Semicond. Process. 2022, 143, 106518.

[24] R. Bano , K. Ayub , T. Mahmood , M. Arshad , A. Sharif , S. Tabassum , M. A. Gilani , Diamond Relat. Mater. 2023, 135, 109826.

[25] A. K. Srivastava , J. Mol. Graphics 2021, 102, 107765. 10.1016/j.jmgm.2020.107765 33069890

[26] H. Park , G. Meloni , ChemPhysChem 2018, 19, 256. 29141115 10.1002/cphc.201701232

[27] A. K. Srivastava , Chem. Phys. Lett. 2018, 695, 205.

[28] A. K. Srivastava , Int. J. Quantum Chem. 2018 118, e25598.

[29] C. Sikorska , N. Gaston , Chem. Phys. 2020, 153, 144301. 10.1063/5.0025545 33086817

[30] A. K. Srivastava , Int. J. Quantum Chem. 2019, 119, e25904.

[31] H. Srivastava , A. K. Srivastava , Front. Phys. 2022, 10, 870205.

[32] M. D. Fryzuk , S. A. Johnson , Coord. Chem. Rev. 2000, 200, 379.

[33] M. P. Shaver , M. D. Fryzuk , Adv. Synth. Catal. 2003, 34, 1061.

[34] R. H. Socolow , Proc. Natl. Acad. Sci. USA 1999, 96, 6001. 10339531 10.1073/pnas.96.11.6001 PMC34219

[35] V. Smil, *The MIT Press, Cambridge, MA*, **2001**.

[36] S.-W. Park , Y. Chun , S. J. Cho , S. Lee , K. S. Kim , J. Chem. Theory Comput. 2012, 8, 1983. 26593832 10.1021/ct300154b

[37] X.-Q. Xiao , Z. Dong , Z. Li , C. Yan , G. Lai , M. Kira , Angew. Chem. Int. Ed. 2016, 55, 3758. 10.1002/anie.201511493 26855202

[38] X. Liu , Y. Ryabenkova , Phys. Chem. Chem. Phys. 2015, 17, 715. 25259662 10.1039/c4cp03568b

[39] Z. Shi , C. Zhang , C. Tang , N. Jiao , Chem. Soc. Rev. 2012, 41, 3381. 22358177 10.1039/c2cs15224j

[40] S. Sui , X. Wang , X. Zhou , Y. Su , S. Riffat , C.-j. Liu , J. Mater. Chem. A 2017, 5, 1808.

[41] T. Punniyamurthy , S. Velusamy , J. Iqbal , Chem. Rev. 2005, 105, 2329. 15941216 10.1021/cr050523v

[42] A. Züuttel , A. Remhof , A. Borgschulte , O. Friedrichs , Philos. Trans. R. Soc. London Ser. A 2010, 368, 3329. 10.1098/rsta.2010.0113 20566514

[43] J. O. Abe , A. P. I. Popoola , E. Ajenifuja , O. M. Popoola , Int. J. Hydrogen Energy 2019, 44, 15072.

[44] K. Mazloomi , C. Gomes , Renewable Sustainable Energy Rev. 2012, 16, 3024.

[45] U. Eberle , M. Felderhoff , F. Sch€uth , Angew. Chem. Int. Ed. 2009, 48, 6608. 10.1002/anie.200806293 19598190

[46] Q. Lai , M. Paskevicius , D. A. Sheppard , C. E. Buckley , A. W. Thornton , M.R Hill , Q. Gu , J. Mao , Z. Huang , H. Liu , Z. Guo , A. Banerjee , S. Chakraborty , R. Ahuja , K. Aguey-Zinsou , ChemSusChem 2015, 8, 2789. 26033917 10.1002/cssc.201500231

[47] R. T. Yang , Carbon 2000, 38, 623.

[48] J. A. Teprovich, Jr. , M. S. Wellons , R. Lascola , S. Hwang , P. A. Ward , R. N. Compton , R. Zidan , Nano Lett. 2012, 12, 582. 22206302 10.1021/nl203045v

[49] P. Mauron , M. Gaboardi , A. Remhof , A. Bliersbach , D. Sheptyakov , M. Aramini , G. Vlahopoulou , F. Giglio , D. Pontiroli , M. Riccòo , A. Züuttel , J. Phys. Chem. C 2013, 117, 22598.

[50] M. Gaboardi , N. S. Amadè , M. Riccòo , C. Milanese , A. Girella , M. Gioventù , F. Fernandez-Alonso , Int. J. Hydrogen Energy 2018 43, 16766.

[51] A. Allangawi , M. A. Gilani , K. Ayub , T. Mahmood , Int. J. Hydrogen Energy 2023, 44, 16663.

[52] A. Allangawi , N. Kosar , K. Ayub , M. A. Gilani , N. H. B. Zainal Arfan , M. H. S. A. Hamid , T. Mahmood , ACS Omega 2023, 41, 37820. 10.1021/acsomega.3c01794 PMC10586255 37867697

[53] C. Mǿller , M. S. Plesset , Phys. Rev. 1934, 46, 618.

[54] M. J. Frisch, G. W. Trucks, H. B. Schlegel, G. E. Scuseria, M. A. Robb, J. R. Cheeseman, G. Scalmani, V. Barone, G. A. Petersson, H. Nakatsuji, X. Li, M. Caricato, A. V. Marenich, J. Bloino, B. G. Janesko, R. Gomperts, B. Mennucci, H. P. Hratchian, et al., Gaussian 16, *Revision C.01. Gaussian, Inc., Wallingford CT* **2019**.

[55] R. Dennington, T. A. Keith, J. M. Millam, *GaussView 6.0. 16. Semichem Inc., Shawnee* Mission **2016**.

[56] A. E. Reed , R. B. Weinstock , F. Weinhold , J. Chem. Phys. 1985, 83, 735.

[57] L. T. Xu , T. H. Dunning , J. Chem. Theory Comput. 2015, 11, 2496. 26575549 10.1021/acs.jctc.5b00104

[58] J. A. Kerr , K. P. Huber , G. Herzberg , Anal. Chim. Acta 1982, 144, 298.

[59] D. R. Lide , CRC Handbook of Chemistry and Physics, CRC Press, Boca Raton, 1996.

[60] E. S. Chen , E. C. M. Chen , J. Phys. Chem. A 2003, 1, 169.

[61] K. M. Ervin , I Anusiewicz , P Skurski , J. Simons and W. C. Lineberge , J. Phys. Chem. A 2003, 41, 8521.

[62] A. G. Samuelson , J. Jabadurai , Resonance 2011, 16, 1152.

[63] S. Sarkar , T. Debnath , A. K. Das , Comput. Theor. Chem. 2021, 1202, 113317.

